# Mini Review of the Use of the Mobile Phone and Its Repercussion in the Deficit of Physical Activity

**DOI:** 10.3389/fpsyg.2019.01307

**Published:** 2019-06-06

**Authors:** María Luisa Zagalaz-Sánchez, Javier Cachón-Zagalaz, María Sánchez-Zafra, Amador Lara-Sánchez

**Affiliations:** Department of Musical, Plastic and Body Expression, University of Jaén, Jaén, Spain

**Keywords:** physical activity, smartphone, addiction, sedentariness, technology

## Abstract

**Background:**

Current technology has the ability to distract and evade its users, this resulting in an addiction or “escape” from the real world. The excessive use of smartphones can cause a decrease in physical activity (PA) for many people prefer to use these devices rather than do the recommended physical exercise.

**Objective:**

The objective of this paper is to analyze the possible relation between the use of smartphones and the reduction in the practice of PA that is reflected in scientific articles.

**Methods:**

The PRISMA statement has been followed for systematic reviews and meta-analyses in order to achieve a correct organization and integrity of the work. Our search for bibliography has been carried out in the WOS and Scopus databases, together with the research domains “Social Sciences Other Topics,” “Education Educational Research,” and “Sport Sciences.” After applying the inclusion criteria, a total of 14 articles were obtained, these forming the base body of this research.

**Results:**

Nine of the fourteen articles show that there is a negative relationship between the smartphone and PA practice. The age groups most studied in relation to this topic range from 13 to 18 and from 19 to 25.

**Conclusion:**

It is our conclusion that the inappropriate use of these mobile devices is associated with low levels of motivation and practice of PA in their users. Similarly, we have emphasized the lack of scientific work dealing with this issue.

## Introduction

The concern about the possible effects of Information and Communication Technologies (ICTs) is no longer new. Current studies allude to the problems caused by the excessive use of small screens, especially smartphones and, therefore, the Internet, as well as addictions to video games..., and also in relation to the dependence they create on users. Since the internet exists, problems related to its use have continuously appeared (Domínguez et al., 2012). Nowadays technology has the ability to distract and evade people from their problems, but it can also result in addictions or become an unhealthy escape from the real world (Sánchez-Zafra et al., 2019).

The speed at which technology advances makes the functionality of smartphones change continuously. Each time, they offer more applications, better camera, more power and memory. They grant a large number of services related to leisure, such as watching television, listening to music or enjoying multiple games. In the same way, they provide current information and allow socializing with other people through social networks (Aranda et al., 2017). This makes them increasingly attractive to children, young and adults, causing certain problems and situations when there is excessive and continuous dependence on these devices (Echeburúa and de Corral, 2010; Alfaro et al., 2015; Rozgonjuk et al., 2018). Over time, smartphones users are normalizing their use, leaving aside the playful part they offer to give them a more professional and personal use. This also occurs in the educational context (Ramírez et al., 2018), where there are more than 80.000 educational Apps.

However, its problematic use can affect various factors in our daily life, causing, for instance, a lack of practice of physical activity (PA) (Sung et al., 2016; Elhai et al., 2017; Ramírez-Granizo et al., 2018). Actions such as sending messages, phoning or surfing the internet are considered sedentary and this can lead to health problems since insufficient levels of moderate and vigorous physical exercise are associated with the increase of obesity. The regular practice of pa is associated with benefits similar to those of some medical treatments. This is why it is an essential activity in our lives (Perea et al., 2015). An addictive use of smartphones is also related to a higher body mass index and a deterioration of physical health, such as loss of vision and musculoskeletal problems (Bell et al., 2014; Kim et al., 2015; Sook and Chun, 2017), in addition to loss of attention and lack of sleep, not to mention -specially among young people- the risk of using it while driving. One of the main factors associated with overuse of smartphones is age. It is the university students, along with the younger ones, who have more normalized its use in their routine (Jasso et al., 2017).

It is scientifically proven that a regular practice of PA is associated with health benefits and that a sedentary lifestyle slows them down, having negative consequences even in active people (Barkley et al., 2016). The first studies focused on the sedentary lifestyle of schoolchildren related it exclusively to the hours they spent watching television but over time it has been also applied to the rest of electronic devices with a screen (Sandercock et al., 2016). As the use of smartphones increases, the practice of PA decreases (Joshi et al., 2016) to such an extent that the fight against sedentary lifestyle and obesity necessarily includes the analysis and harm of the abusive use of smartphones. Venkatesh et al. (2017) confirm that adults addicted to these devices do less physical exercise if compared to those who are not, and Samaha and Hawi (2017) obtain the same results in a population of children. Sedentary lifestyles, motivated among other reasons by the excessive use of smartphones, are becoming one of the main concerns of society (Glynn et al., 2013).

Despite these disadvantages, the use of smartphones does not necessarily lead to sedentary actions. In fact, there are many applications in the market helping us in our practice of physical activity. Some remind users of the moment when they have to do exercise or what kind of activity they have to do that day (Patel et al., 2018). Likewise, the research by Shen et al. (2018) states that the people who practice PA most are those who most consult their smartphones for health-related issues.

Our main objective is to conduct a systematic review of scientific literature focused on the use of the smartphone and its relationship with the practice of PA in populations of different ages without diseases.

## Methodology

The PRISMA statement has been followed for systematic reviews and meta-analyses (Hutton et al., 2016) in order to achieve a correct organization and integrity of the work.

### Procedure and Literature Search Strategy

The literature review of the articles was carried out in November and December of 2018, paying special attention to those relating the excessive use of the smartphone with the amount of PA in the last decade.

The Web of Science (WOS) and Scopus databases have been used to search for the articles. The articles were searched using the following keywords: “mobile phone,” “physical activity” and “the Boolean operator.” Subsequently, the search was refined taking into account only the articles published in the research areas “Social Sciences Other Topics,” “Education Educational Research,” and “Sport Sciences,” leaving a total of 271 documents.

### Inclusion Criteria

The inclusion criteria were established: (1) articles that relate the use of smartphones to the practice of PA, (2) use that is given to mobile phones, and (3) whether the research is cross-sectional or longitudinal.

### Study Selection and Data Collection Process

The first part of the search initially obtaining an amount of 1.908 articles. For the application of the first inclusion criteria, a first reading of the title and summary of the 271 selected papers was made. In a following stage, a second deeper reading was necessary to apply the rest of the inclusion criteria. After the application of these criteria, a total of 257 articles were eliminated.

### Population and Scientific Literature Sample

The population of this study is set at 271 articles extracted from the WOS and Scopus databases. After applying the inclusion criteria, the study sample is refined to 14 scientific publications.

## Results

### Evolution of the Scientific Production

Considering the selected research areas and the inclusion criteria, 271 articles related to the subject under discussion have been published in the last decade. 14 works have been selected to constitute the body of the investigation.

### Data From the Studies Selected for the Systematic Review

For the selection of the articles and their coding (Table 1), the following data have been taken into account: (1) authors and year of publication, (2) country where the work has been carried out, (3) type of research carried out, (4) size of the sample, (5) sample age, (6) instruments, (7) variables that have been measured, and (8) relation between PA and the use of smartphones, understanding that the latter is positive when there is an active use of these devices whereas it is negative when it is a passive or sedentary one.

**Table 1:** Basis of the study.

Authors and year	Country	Kind of investigation	Age of the Sample	Instrument^∗^	Relation PA-smartphone
Arora et al. (2013)	United Kingdom	Cross	11–18 years old	Stadiometer Scales *Ad hoc* TUQ	Positive
Barkley and Lepp (2016)	United States	Cross	18–34 years old	GLTEQ *Ad hoc* *Ad hoc*	Negative
Beltrán-Carrillo et al. (2016)	Spain	Cross	15–16 years old	*Ad hoc*	Positive
Delfino et al. (2018)	Brazil	Cross	10–17 years old	*Ad hoc*	Negative
Haug et al. (2015)	Switzerland	Cross	15-older than 21	*Ad hoc* SAS-SV *Ad hoc*	Negative
Kim et al. (2015)	Korea	Cross	19–25 years old	SAPS Pedometer 320 Body Composition Analyzer Anthropometer	Negative
Lane et al. (2014)	Ireland	Longitudinal	9 years old	*Ad hoc*	Negative
Lepp et al. (2013)	United States	Cross	University students	SESEB *Ad hoc* *Ad hoc*	Negative
Mojica et al. (2014)	United States	Cross	11–14 years old	YRBSS *Ad hoc*	Positive
Morita et al. (2016)	Japan	Cross	12–13 years old	PA *Ad hoc*	Negative
Sevil et al. (2018)	Spain	Cross	Average age: 13 years old	*Ad hoc*	Negative
Shen et al. (2018)	Hong Kong	Cross	18-older than 65	HIE HB	Positive/Negative
Silva et al. (2017)	Portugal	Cross	13–19 years old	*Ad hoc* *Ad hoc*	Negative
Zach et al. (2016)	Israel	Longitudinal	16–18 years old	*Ad hoc* *Ad hoc*	Positive

*^∗^TUQ, technology use questionnaire; GLTEQ, godin leisure-time exercise questionnaire; SAS-SV, smartphone addiction scale short version; SAPS, smartphone addiction proneness scale; SESEB, self-efficacy survey for exercise behaviors; YRBSS, youth risk behavioral surveillance system; HIE, health information exposure; HB, health behaviors.*

The reviewed articles are spread across countries all over the world without being concentrated in any particular geographical area. The majority of the works belong to a cross sectional paradigm. With respect to the instruments, as highlighted, there is no validated universal questionnaire that measures the relation between the use of smartphones and their relationship with the practice of PA, since our approach reveals that each author has chosen a different questionnaire for their research, many of them being of own elaboration. Regarding the relationship between PA and the use of smartphones, most of the works speak of a negative, passive use, in which the time of physical exercise is considerably reduced. Others point out a positive use, focusing on applications or ways to encourage the practice of PA thanks to smartphones.

**FIGURE 1:**
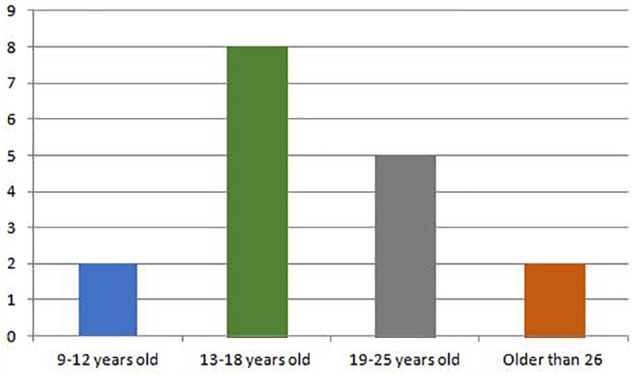
Distribution of selected articles according to age.

Figure 1 shows the distribution of the articles that make up the sample according to the age of the participants. Some of the studies are framed in more than one age group because they used participants of different ages. It can be seen that most of the articles focus on an age range between 13 and 18 years old (*N* = 8), followed by 19 to 25 years old (*N* = 5). The least studied ages are those under 12 (*N* = 2) and those above 26 (*N* = 2).

## Discussion

Most of the papers presented in the Results section share the view that there is nowadays an excessive use of smartphones among children, adolescents and adults. It is the youngest who spend more hours in front of the screens and those who have more problems and addictions (Haug et al., 2015).

In the study by Lane et al. (2014), there arises the discussion on the recommendation made by the American Academy of Pediatrics (2001) about not using smartphones for more than 2 h a day. This recommendation is not met by the sample of the articles studied, according to which children between 6 and 12 spend an average of 40 h a week talking, writing text messages, listening to music and surfing the Internet. Most of them can send text messages through the internet thanks to the data plans they have (Mojica et al., 2014). According to Sudan et al. (2016) children born in the last 20 years have been exposed to these devices since birth and, therefore, their relationship with them is different and cannot be compared with that of adults. Children are continually watching videos, playing games or talking on WhatsApp. They live attached to the smartphone.

Sevil et al. (2018) show that adolescents spend an average of 407 min a day (6.7 h) with different technological devices, smartphones being the second most used to communicate with others, for example, through WhatsApp. As with children, these adolescent users have in many cases flat data rates, which allow them to use smartphones anywhere and anytime, progressively increasing the time spent on them (De-Sola et al., 2016). Preadolescents and adolescents spend more time with smartphones, and therefore have a more sedentary behavior than younger children (Joshi et al., 2016).

Likewise, the research carried out by Lepp et al. (2013) and Barkley and Lepp (2016) show that the average number of minutes per day spent by university users is 300 and 380, respectively. Horwood and Anglim’s (2018) survey showed that 54% of adults used smartphones when they should be doing other things and that 34% lost sleep hours when using them. 65% of respondents confirm that they have used smartphones for a longer time than they had originally planned. In university classes it is normal to see students using the smartphone instead of attending to the teacher. Even so, older people use less smartphones than younger ones (Barkley and Lepp, 2016).

The majority of studies imply that there are no significant differences in the use of the smartphone as far as gender is concerned, but Beltrán-Carrillo et al. (2016) state women use these devices more frequently, coinciding with other studies such as Joshi et al. (2016) or Pedrero-Pérez et al. (2018), who explain that women use instant messages and social networks more, men preferring the internet and video games.

As confirmed by most studies, the time users spend with smartphones is associated with sitting. Those who use the smartphone less spend less time sitting than those who have a more continuous use of it (Barkley and Lepp, 2016). The muscle mass of people with addiction to smartphones was much lower than that of the more moderate users. The opposite occurs with fat mass, since the more a smartphone is used, the more fat mass users have (Kim et al., 2015). Furthermore, the misuse of smartphones is associated with a greater proclivity to suffer body aches in the back, neck (today’s boys are known as the generation of crouched heads), wrists, hips and knees (Silva et al., 2017).

The relation between the misuse of smartphones and the practice of PA is obvious. There are authors who speak of a negative relation, arguing that the excessive use of smartphones produces a sedentary lifestyle and inactivity, leading to a reduction in the time devoted to physical exercise. Nowadays, the majority of the population prefers to use the smartphone instead of going for a walk or doing some kind of physical exercise. The first thing young people do when they leave class and adults do when they leave work is to take the smartphone. Delfino et al. (2018) observed that users who spent more than 2 h using smartphones were physically less active than others. These researchers suggest that the practice of low intensity PA can help to reduce the time spent on these devices. Additionally, sedentary behaviors should be discouraged and foster a change from moderate physical exercises to vigorous ones (Morita et al., 2016).

The same users are aware, in many cases, that smartphones can interfere with their PA and many of them say they leave their smartphone off in order to be more active and reduce sedentary behavior (Lepp et al., 2013). On the other hand, it is found that those people who do more moderate and vigorous PA are the ones who use the smartphone most to consult health issues (Shen et al., 2018).

Conversely, smartphones can also be used to reduce sedentary levels and increase PA. It should not be forgotten that they are small devices that can be carried anywhere and used in movement (Arora et al., 2013). It is important to widespread the use of ICTs in PA classes in order to promote knowledge and increase healthy lifestyles in terms of physical exercise (Zach et al., 2016). There are a multitude of games and applications that can be used to encourage PA practice. It is important for children to know them.

Similarly, there are several studies that show that the use of messages through the smartphone aimed at enhancing the levels of PA in users is effective, since sedentary lifestyles can be reduced through reminders on the smartphone to get up when the user has been sitting for a long time, to go for a walk or do any simple exercise (McCoy et al., 2017; Dunning et al., 2018). Through virtual reality, some aspects related to PA can be improved more effectively, such as spatial orientation (Gómez-García et al., 2018).

## Conclusion

Our analysis reveals that the number of studies that address the issue of the influence of smartphones in the practice of PA is scarce and that, in spite of having considered a whole decade in our approach, they were all published only in the last 5 years. This question has been raised globally, finding that the investigations have been carried out in different countries, inferring that it is a subject of special relevance and international significance. At the same time, we have highlighted that most of them are cross-sectional studies.

All the stages have been considered, from children to adults, and each of them shows that the inappropriate use of smartphones reduces the practice of PA, since these devices are preferred. On the other hand, some works claim that a suitable use of smartphones can foster the recommended daily exercise, for example, through applications or messages that remind and encourage the activities to be carried out.

Regarding the limitations of our study, we have only covered a decade and, consequently, this research is likely to be developed and extended to see if more articles investigating this topic have been published since the appearance of smartphones.

## Author Contributions

MZ-S, JC-Z, MS-Z, and AL-S contributed to the conception and design of the revision. All authors wrote some part of the manuscript and all reviewed the manuscript.

## Conflict of Interest Statement

The authors declare that the research was conducted in the absence of any commercial or financial relationships that could be construed as a potential conflict of interest.
